# Spontaneous Bacterial Peritonitis Beyond the Usual Suspects: Case Series of *Salmonella* and *Brucella* With Comprehensive Literature Review

**DOI:** 10.1002/ccr3.9647

**Published:** 2025-02-26

**Authors:** Hussam Almasri, Elhassan Mahmoud, Ashraf I. Ahmed, Hamad A. Alkorbi, Aiman Ahmed, Shahem Abbarh, Bisher Sawaf

**Affiliations:** ^1^ Internal Medicine Department University of North Dakota Fargo North Dakota USA; ^2^ Department of Internal Medicine Hamad Medical Corporation Doha Qatar; ^3^ College of Medicine, QU Health Qatar University Doha Qatar; ^4^ Department of Internal Medicine MedStar Health Baltimore Maryland USA; ^5^ Department of Internal Medicine University of Toledo Medical Center Toledo Ohio USA

**Keywords:** atypical pathogens, *brucella*, liver cirrhosis, *salmonella*, spontaneous bacterial peritonitis (SBP)

## Abstract

While *Escherichia coli* , *Streptococcus*, and *Klebsiella* species traditionally account for most cases of spontaneous bacterial peritonitis (SBP), atypical pathogens can be found. *Salmonella*, typically known for causing gastroenteritis, rarely manifests as SBP, while *Brucella*, a zoonotic pathogen, presents a unique challenge in the context of peritoneal infection. In this report, we present two cases of SBP caused by these atypical organisms. The first case involved a 58‐year‐old female with a history of hepatitis C‐related chronic liver disease and cryoglobulinemic vasculitis, who presented with SBP and hepatorenal syndrome‐associated acute kidney injury. *Salmonella* was identified in both ascitic fluid and blood cultures, but despite appropriate treatment, the patient experienced a fatal deterioration. The second case features a 52‐year‐old male with type 2 diabetes mellitus and newly diagnosed schistosomiasis, who presented with findings of decompensated liver cirrhosis and SBP. *Brucella* was isolated from ascitic fluid cultures, and the patient responded well to treatment. We aim to highlight the need for early recognition of atypical causes of SBP to improve management and outcomes.

**Summary**

- These two cases emphasize the importance of considering atypical pathogens such as *Salmonella* and *Brucella* in the differential diagnosis of spontaneous bacterial peritonitis (SBP), particularly in patients with specific risk factors such as immunosuppression or exposure to endemic areas.
- Early recognition, culture‐based identification, and timely antibiotic adjustments are crucial in improving patient outcomes, highlighting the need for vigilance in the management of SBP beyond the more commonly recognized causative agents.

## 1 Introduction

Spontaneous bacterial peritonitis (SBP) poses a formidable challenge in the management of patients with advanced liver disease and ascites, representing a significant cause of morbidity and mortality in this population [1]. The aforementioned clinical entity is defined as an infection of the ascitic fluid without an identifiable intra‐abdominal source. SBP emerges from a complex interplay of factors, foremost among them being bacterial translocation, which facilitates the migration of gut bacteria into the peritoneal cavity [2]. While *Escherichia coli* , *Streptococcus*, and *Klebsiella* species traditionally account for the majority of SBP cases, atypical pathogens such as *Salmonella* and *Brucella* pose diagnostic and therapeutic dilemmas [3]. *Salmonella*, typically known for causing gastroenteritis, occasionally manifests as SBP, while *Brucella*, a zoonotic pathogen, presents a unique challenge in the context of peritoneal infection. Prompt recognition and initiation of empiric antibiotic therapy are paramount in suspected SBP to mitigate its associated morbidity and mortality. However, challenges persist in cases where conventional antibiotic regimens falter, necessitating a meticulous diagnostic approach to uncover the underlying etiology and tailor therapy accordingly. In this present study, we present two cases of SBP caused by *Salmonella* and the other by *Brucella* species. Through the detailed examination of these cases, we aim to highlight the importance of vigilance, early intervention, and a multidisciplinary approach in navigating the diagnostic and therapeutic challenges posed by atypical presentations of SBP.

## 2 Case Presentation

### 2.1 Case I. SBP—*Salmonella*

#### 2.1.1 Case History and Examination

A 58‐year‐old Middle Eastern woman residing in Qatar presented to the Emergency Department with a two‐day history of diffuse abdominal pain and distension. The pain was associated with fever and loose, non‐bloody motions. She denied vomiting or a skin rash. Her medical history includes controlled diabetes and hypertension. Notably, 2 months prior to presentation, she was found to have hepatitis C‐related liver cirrhosis, with a Child‐Pugh score of 8 (Class B) and a Model for End‐stage Liver Disease (MELD) score of 13. At that time, she was also diagnosed with cryoglobulinemic vasculitis and glomerulonephritis and was treated with prednisolone and rituximab. Additionally, she was started on sofosbuvir/daclatasvir. On presentation, the patient was febrile, with a heart rate of 125 beats per minute and a blood pressure of 72/42 mmHg. The abdomen was soft but distended and diffusely tender, with a positive shifting dullness. Other examinations were unremarkable.

#### 2.1.2 Investigation and Diagnosis

Laboratory tests were significant for normocytic anemia with a hemoglobin of 8 g/dL and elevated inflammatory markers. There was also a new worsening in her renal function with a creatinine of 128 μmol/L and urea of 8.7 mmol/L. Liver aminotransaminases were normal. Ascitic fluid analysis revealed more than 37,000/μL white blood cell count (WBC), 91% being neutrophils. The serum ascites albumin gradient was 9.3 g/dL. Cultures from ascitic fluid, blood, and stool were obtained. An abdomen ultrasound showed an increase in the amount of ascitic fluid, which highly suggests a provisional diagnosis of SBP.

#### 2.1.3 Treatment, Outcome, and Follow‐Up

The patient was admitted to the medical intensive care unit and resuscitated with intravenous fluids, hydrocortisone, and vasopressors. Empirical treatment with piperacillin/tazobactam was initiated. Subsequently, cultures from blood, ascitic fluid, and stool confirmed the presence of group G2 *Salmonella*, sensitive to ceftriaxone. Unfortunately, despite aggressive management, the patient's condition continued to deteriorate with significant acute kidney injury, and she passed away 1 week after admission.

### 2.2 Case II. SBP—*Brucella*

#### 2.2.1 Case History and Examination

A 52‐year‐old African man residing in Qatar presented to the Emergency Department with a fever persisting for 5 days. He also complained of diffuse abdominal pain and non‐bloody loose motion. Additionally, he reported coughing with the production of yellow sputum and right‐side pleuritic chest pain. His past medical history is significant only for controlled diabetes mellitus managed with oral antihyperglycemic agents. He works on farmland and has direct contact with camels. He is a non‐smoker and denied alcohol consumption. On examination, vital signs were significant for fever and tachycardia, otherwise within normal limits. Chest examination revealed decreased breath sounds with coarse crackles in the right middle zone. The abdomen was soft but diffusely tender, with positive shifting dullness and splenomegaly.

#### 2.2.2 Investigation and Diagnosis

Laboratory tests indicated elevated inflammatory markers, normocytic anemia with a hemoglobin level of 10.7 g/dL, and a platelet count of 72 × 10 μL [3]. Liver function tests showed aspartate aminotransferase (AST) at 152 U/L, alanine aminotransferase (ALT) at 80 U/L, alkaline phosphatase (ALP) at 592 U/L, and total bilirubin at 36 μmol/L, primarily elevated direct bilirubin. Other labs showed normal creatinine, mild hyponatremia, and an INR of 1.3. Chest X‐ray revealed consolidation of the right middle lobe. Abdominal ultrasound revealed moderate ascites, splenomegaly, and a shrunken liver with cirrhotic features, periportal fibrosis, and portal hypertension. Ascitic diagnostic paracentesis revealed a WBC count of 1050 μL, with 62% neutrophils, and a serum‐ascites albumin gradient of 1.6 g/dL. The patient was admitted with a provisional diagnosis of community‐acquired pneumonia and SBP.

#### 2.2.3 Treatment, Outcome, and Follow‐Up

The patient was initially started on ceftriaxone and azithromycin. However, due to a lack of clinical improvement on day 3, antibiotics were escalated to piperacillin/tazobactam. On day 5 of admission, blood and ascitic fluid cultures returned positive for *Brucella*. Treatment was adjusted to include doxycycline plus rifampin. Chronic liver disease evaluation revealed positive *Schistosoma* antibodies and esophageal varices necessitating band ligation. The patient was diagnosed with decompensated liver cirrhosis (Child Class B and MELD score of 12) secondary to schistosomiasis and received praziquantel. Furosemide, spironolactone, and propranolol were also initiated. Subsequently, he demonstrated significant improvement and was discharged with a follow‐up in infectious disease and gastroenterology clinics.

## 3 Discussion

SBP is a life‐threatening complication that most commonly affects patients with liver cirrhosis. A diagnosis of SBP is made if the absolute polymorphonuclear cell count in ascitic fluid is ≥ 250 cells/mm^3^ in the absence of secondary causes of peritonitis. It is most commonly caused by *E. coli* , *Streptococcal* species, and *Klebsiella pneumoniae* [4]. However, in this case series, we presented two cases of SBP that are caused by *Salmonella* and *Brucella*, highlighting that these atypical organisms can be causative agents which usually lead to significant challenges in management due to their rarity and different antibiotic coverage.

The literature on SBP caused by *Salmonella* and *Brucella* is sparse, with very few cases reported for each organism. In reviewing the literature (Tables 1 and 2), we can see that SBP caused by these atypical organisms can occur in patients with specific risk factors, including immunosuppression, travel to or residence in endemic areas, and contact with animals or consumption of unpasteurized dairy products.

**TABLE 1:** Patients with SBP caused by *Salmonella* in the literature.

Age	Sex	Comorbidities	Recent immunosuppressant use	Organism	Treatment	Outcome	References
40	Female	Alcoholic liver cirrhosis	Yes (prednisolone)	*Salmonella typhimurium*	Ceftriaxone initially; switched to meropenem; narrowed back to ceftriaxone after sensitivity testing	Stabilized by day 12, discharged with no recurrence after 4 months, required monthly paracentesis (~5 L drained each time)	[5]
60	Male	Alcoholic liver cirrhosis, hepatitis C	No	*S. typhimurium*	Ceftriaxone; switched to piperacillin‐tazobactam after sensitivity testing	Discharged on ciprofloxacin prophylaxis, no relapse	[8]
63	Male	Liver cirrhosis, hepatitis C, metastatic hepatocellular carcinoma	Yes (lenvatinib, sorafenib)	*S. enterica*	Ceftriaxone	Discharged on trimethoprim/sulfamethoxazole prophylaxis	[9]
57	Female	Diabetes mellitus, pulmonary tuberculosis, chronic liver disease	No	*S. typhimurium*	Cefotaxime	Recovered completely; sterile repeat cultures after 5 days	[10]
47	Female	Liver cirrhosis	Unknown	*S. typhimurium*	Cefotaxime	Died 20 days after admission despite regression of hepatic encephalopathy and fever	[11]
61	Female	Cryptogenic liver cirrhosis, diabetes mellitus type 2, hypertension, B‐cell non‐hodgkin lymphoma	Yes (rituximab, dexamethasone)	Group B *Salmonella*	Ceftriaxone	Recovered after 1 week of treatment; followed by oral ciprofloxacin	[12]
51	Male	Alcoholic cirrhosis, chronic colitis	Yes (5‐aminosalicylate)	*S. dublin*	Cefotaxime	Developed renal failure; died 10 days later	[13]

**TABLE 2:** Patients with SBP caused by *Brucella* in the literature.

Age	Sex	Occupation	History of unpasteurized dairy product consumption	Comorbidities	Treatment	Outcome	References
69	Male	Stockbreeder	No	None	Cefotaxime (initial) and vancomycin (for spondylodiscitis); switched to streptomycin, doxycycline, and rifampicin	Improved after 10 days; ascites resolved within a month; no recurrence after 2 years	[7]
47	Male	Businessman	No	Hepatitis C, type 2 diabetes mellitus	Imipenem/cilstatin (initial); switched to doxycycline and rifampin	Improved after therapy change; discharged after 4 weeks, continued antibiotics for 8 weeks	[14]
65	Female	Unknown	Yes	Liver cirrhosis, chronic hepatitis C	Rifampin and doxycycline; later switched to streptomycin and TMP‐SMX after developing hyperbilirubinemia and prolonged prothrombin time on day 9	Became afebrile on day 6; streptomycin stopped on day 21 and was discharged on TMP‐SMX and ciprofloxacin; stopped antibiotics on day 42; no recurrence after 1 year follow‐up	[15]
60	Female	Unknown	No	Liver cirrhosis, hepatitis C	Ciprofloxacin and rifampin	Improved and became after 1 week	[16]
65	Male	Unknown	No	Liver cirrhosis, hepatitis B	Ceftriaxone (initial); switched to doxycycline, ceftriaxone, and ofloxacin after *Brucella* was confirmed	Hepatocellular carcinoma was diagnosed during hospitalization; died on day 10 due to hepatic failure	[17]
63	Male	Unknown	Unknown	Diabetes mellitus, cryptogenic liver cirrhosis	Rifampin and doxycycline	Improved and became afebrile after 1 week; ascites resolved after 2 months	[18]

In our first case, the patient's liver cirrhosis combined with her history of immunosuppressive therapy likely contributed to the development of *Salmonella*‐induced SBP. A review of similar cases in the literature shows that in 4 out of 7 cases of SBP caused by *Salmonella*, the patients had recent history of immunosuppressant use, with an additional patient having diabetes mellitus which could have contributed to the disease by inducing an immunocompromised state. Furthermore, it is important to note that cirrhosis by itself can cause an immunocompromised state as a result of a phenomenon known as cirrhosis‐associated immune dysfunction that leads to immunodeficiency [5]. Therefore, the use of immunosuppressants or the presence of another comorbid condition that leads to an immunocompromised state most likely acts synergistically with the cirrhosis‐associated immune dysfunction, leading to further suppression of the immune system, consequently increasing the risk of *Salmonella*‐induced SBP. Moreover, the outcomes of *Salmonella*‐induced SBP vary widely, with some patients responding well to targeted antibiotic therapy and others, as in our case, experiencing rapid clinical deterioration and death despite appropriate treatment. This underscores the critical importance of early detection and aggressive management in these patients.

In the second case, the patient presented with *Brucella*‐induced SBP, an extremely rare manifestation of brucellosis. Brucellosis is typically associated with occupational exposure to animals or consumption of unpasteurized dairy products. It typically presents with flu‐like symptoms, night sweats, undulant fever, lymphadenopathy, arthralgias, and epididymo‐orchitis [6]. However, although rare, brucellosis can affect any organ, including neurologic, cardiovascular, pulmonary, and intra‐abdominal involvements. While SBP is generally associated with underlying liver disease, *Brucella*‐induced SBP can occur even in patients without pre‐existing liver conditions, as in our patient and Makaritsis et al.'s [7] patient. Therefore, a high index of suspicion for diagnosis is needed, particularly in patients with risk factors such as those seen in our patient. Additionally, there are no clear recommendations regarding the treatment of *Brucella*‐induced SBP, likely due to its rarity. The literature of published cases suggests that the combination of doxycycline and rifampin, as used in our case, is effective in treating SBP caused by *Brucella*, leading to favorable outcomes when initiated promptly. However, others have proposed treatment with streptomycin in addition to doxycycline and rifampin for a period of at least 3–4 months [7].

## 4 Conclusion

In conclusion, while SBP is most commonly caused by enteric gram‐negative bacteria, including *E. coli* , our cases and the existing literature highlight importance of being aware of atypical organisms like *Salmonella* and *Brucella*. The consideration of these pathogens in the differential diagnoses, particularly in patients with specific risk factors or epidemiological exposures, can significantly improve outcomes. Early identification through culture and sensitivity testing, followed by appropriate antibiotic adjustments, is essential in managing these cases. Further case reports and studies are necessary to better understand the optimal management strategies and the outcomes for SBP caused by these organisms.

## Author Contributions

**Hussam Almasri:** conceptualization, methodology, validation, visualization, writing – original draft, writing – review and editing. **Elhassan Mahmoud:** conceptualization, methodology, validation, visualization, writing – original draft, writing – review and editing. **Ashraf I. Ahmed:** conceptualization, validation, visualization, writing – original draft, writing – review and editing. **Hamad A. Alkorbi:** conceptualization, validation, visualization, writing – original draft, writing – review and editing. **Aiman Ahmed:** conceptualization, validation, visualization, writing – original draft, writing – review and editing. **Shahem Abbarh:** conceptualization, validation, visualization, writing – original draft, writing – review and editing. **Bisher Sawaf:** conceptualization, supervision, validation, visualization, writing – original draft, writing – review and editing.

## Ethics Statement

The article was approved by the Institution Review Board at Hamad Medical Corporation.

## Consent

A written informed consent was obtained from the patients for the publication of all images, clinical data, and other data included in the manuscript. All identifying information has been removed.

## Conflicts of Interest

The authors declare no conflicts of interest.

## Data Availability

The data that support the findings of this study are available from the corresponding author upon reasonable request.
